# Turbine Sound May Influence the Metamorphosis Behaviour of Estuarine Crab Megalopae

**DOI:** 10.1371/journal.pone.0051790

**Published:** 2012-12-11

**Authors:** Matthew K. Pine, Andrew G. Jeffs, Craig A. Radford

**Affiliations:** Leigh Marine Laboratory, University of Auckland, Warkworth, Auckland, New Zealand; University of Connecticut, United States of America

## Abstract

It is now widely accepted that a shift towards renewable energy production is needed in order to avoid further anthropogenically induced climate change. The ocean provides a largely untapped source of renewable energy. As a result, harvesting electrical power from the wind and tides has sparked immense government and commercial interest but with relatively little detailed understanding of the potential environmental impacts. This study investigated how the sound emitted from an underwater tidal turbine and an offshore wind turbine would influence the settlement and metamorphosis of the pelagic larvae of estuarine brachyuran crabs which are ubiquitous in most coastal habitats. In a laboratory experiment the median time to metamorphosis (TTM) for the megalopae of the crabs *Austrohelice crassa* and *Hemigrapsus crenulatus* was significantly increased by at least 18 h when exposed to either tidal turbine or sea-based wind turbine sound, compared to silent control treatments. Contrastingly, when either species were subjected to natural habitat sound, observed median TTM decreased by approximately 21–31% compared to silent control treatments, 38–47% compared to tidal turbine sound treatments, and 46–60% compared to wind turbine sound treatments. A lack of difference in median TTM in *A. crassa* between two different source levels of tidal turbine sound suggests the frequency composition of turbine sound is more relevant in explaining such responses rather than sound intensity. These results show that estuarine mudflat sound mediates natural metamorphosis behaviour in two common species of estuarine crabs, and that exposure to continuous turbine sound interferes with this natural process. These results raise concerns about the potential ecological impacts of sound generated by renewable energy generation systems placed in the nearshore environment.

## Introduction

Underwater tidal turbine technology has advanced at a rapid rate due to increasing commercial interest across many countries. This is the result of a widely recognised need to shift energy production from fossil fuels to renewable sources in order to limit further anthropogenically induced climate change [1]–[3]. Tidal power generation is an emerging renewable energy technology, and many wind turbines are already in place within coastal waters of numerous countries [4] and a few pilot projects on underwater tidal turbines [1], [5], [6].

The advantages of renewable energy generation are not in doubt; however, locally the environmental impacts can be significant and need to be carefully considered [1]. While wind turbine farms in coastal waters are well established in Northern Europe and their environmental impacts have been studied to some extent, underwater tidal turbines are still in their infancy and their impacts are largely unknown [1]. The impact of anthropogenic underwater sound on marine life is of growing concern, with an increasing body of evidence indicating negative impacts [1], [7], [8]. The sound generated during the construction and installation of turbine farms has already been identified as being of concern as pile-driving has been observed to directly impact cetaceans and fishes [1], [8]. Very little is understood about the operational sound of underwater tidal turbines and further research is required before drawing conclusions on how their sound will influence marine life [1]. The underwater sound from tidal turbines will be influenced by several factors, including blade and turbine design, tidal flow velocity, depths, bottom substrate, gearboxes, and weather [9]. Similarly, wind speeds and turbine technology also influences the sound generated from operating offshore wind turbines [10]. Therefore, the sound generated and its impacts will be specific to sites and generating devices [9]. The sound from an operating ‘*SeaFlow*’ tidal turbine has been measured to have a source level of approximately 175 dB re 1 µPa @ 1 m with peak intensities at 0.1, 0.8, 2, 5 and 8 kHz [11] at a maximum tidal flow of approximately 3 ms^−1^ [9], [12]. Offshore wind turbines have been found to have an operational underwater source level of 154 dB re 1 µPa @ 1 m at a wind speed of 13 m s^−1^ [10]. These source levels are significantly louder than the ambient underwater sound levels commonly encountered in coastal waters. Consequently, the addition of these anthropogenic sound sources are likely to result in the masking of underwater ambient sound for organisms that rely on acoustic communication or natural acoustic cues within this frequency range [7].

The life history of brachyuran crabs typically involves a planktonic larval stage that ends with a post-larva, or megalopa, that actively swims to find suitable benthic habitat in which to settle and develop into a reptant juvenile [13]–[19]. To help ensure megalopae settle in a suitable location, they have evolved the ability to detect and orient toward physical and chemical cues associated with their preferred benthic habitats [20]–[22]. Once megalopae encounter their preferred habitat, settlement and subsequent metamorphosis from the megalopa to juvenile is often instigated by a combination of several physical and chemical cues, which can include acoustic cues [19], [22]–[24].

The duration of the megalopal stage can be relatively plastic and may depend on the presence or absence of several settlement cues [19], [25], [26]. For example, in the presence of estuarine water the megalopae of the blue crab, *Callinectes sapidus*, decrease their time to metamorphosis (TTM), i.e., the time taken for the larva to moult from a megalopa to the first instar juvenile crab [27]. However, delaying metamorphosis for too long (beyond a specific temporal threshold) may result in the death of the megalopa, or result in spontaneous metamorphosis of the larva followed by poor subsequent post-settlement growth [19], [22], [23], [25], [26], [28]. These temporal thresholds are typically determined in the laboratory by rearing megalopae in control treatments of “untainted” seawater [19]. Depending on the species, the TTM of megalopae can typically be shortened by approximately 15 to 60% upon exposure to appropriate settlement cues [19], [23]. For example, when subjected to reef sound the megalopae of five common coastal species of reef-dwelling brachyuran crabs all accelerated their physiological development and TTM was reduced by between 34 and 60% [19]. These results suggest that natural underwater sound plays an important role in the metamorphosis of brachyuran crabs, especially coastal reef dwelling species. However, no data have been published to suggest the same responses are seen in estuarine species when exposed to estuarine sound. The characteristics of underwater sound that are responsible for expediting metamorphosis in crabs are unknown at this, but may involve sound intensity, frequency composition, or temporal variability in both frequency and intensity, or any combination of these acoustic characteristics. Furthermore, it is possible that other sources of underwater sound may elicit or interfere with the normal metamorphosis response of megalopae in relation to natural acoustic cues. The biological effects of anthropogenic sound in the underwater environment have become of increasing interest in response to rising levels of anthropogenic sound in coastal and ocean waters [7], [8].

The effects of anthropogenic sound on marine mammals and adult fishes have been well studied [7], [8]. However, very few studies have dealt with larvae of marine organisms, and none have investigated the effect of anthropogenic sound on the settlement and metamorphosis of crustacean larvae. Furthermore, no experimental data have been published which investigates the metamorphosis response of estuarine crab megalopae to ambient mudflat sound or the possible effect of tidal and wind turbine sound on their metamorphosis behaviour.

Therefore, the aim of the current research was three-fold: (1) to determine the metamorphosis response of the megalopae of two common estuarine crabs in New Zealand, *Austrohelice crassa* and *Hemigrapsus crenulatus*, to natural ambient estuarine sound; (2) determine whether the underwater sound emitted from tidal and wind turbines influences the metamorphosis response of the crab megalopae, and; (3) attempt to identify which characteristics of turbine sound are responsible for eliciting any observed changes in metamorphosis behaviour of the megalopae.

## Methods

### Ethics statement

This study was carried out under the University of Auckland Animal Ethics Committee approval numbers R701 and R948.

### Sourcing crab megalopae for behavioural assays

All experiments were completed between May 2011 and May 2012 using light traps to capture pelagic crab megalopae [19]. Up to three traps were deployed at night at the same location along the coast at Leigh, in north-eastern New Zealand. The following morning the captured megalopae were placed in containers filled with saltwater and transported back to the laboratory where they were identified, counted and sorted by settlement stage. Only intermoult pre-settlement (i.e., natant and actively swimming) megalopae were selected for use in each experimental assay. If a trap contained large planktivorous fishes, megalopae were not used as they may have altered behaviour due to being in the presence of a predator [19], [29]. Selected megalopae were contained in UV treated and filtered (40 µm) seawater in the laboratory, under natural photoperiod and ambient temperature until experiments begun at 17∶00 hrs on the day of capture [19].

### Behavioural assays

Each experimental assay consisted of silent (control, as no settlement cues) and sound treatments, all of which were contained within the same laboratory, but acoustically isolated by the use of foam rubber mats. The absence of any acoustic signals transmitting from the sound treatments to the silent treatments was confirmed by recording with a calibrated omnidirectional hydrophone (HTI-96 min, High Tech Inc., USA).

Experimental replicates consisted of a water bath (used to maintain constant water temperature) which was used to hold up to ten 250 mL plastic vials with sealed lids that each contained an individual megalopa in 230 mL of 1 µm filtered and UV treated seawater. Any one treatment consisted of three replicates and each replicate contained at least seven megalopae, i.e., at least 21 megalopae per treatment. The number of treatments per experiment varied between two and four depending on the availability of megalopae from the light traps.

Megalopae require roughened substrates to settle [19] and thus each vial had a roughened base to simulate a chemically inert settlement substrate for megalopa. Both the silent and sound treatments contained a Phillips loudspeaker inside a water tight plastic bag on the bottom of the water bath [19]. For the sound treatments, a MP3 player was connected to a Phillips SBA1500 amplifier and speaker to continuously playback a 10 min loop of recorded turbine or mudflat sound into the water bath which was also transmitted through the acoustically transparent plastic vials holding the megalopae.

When sufficient megalopae (at least 21) of the same species and similar settlement stage were captured in the light traps, an individual larva was placed in each 250 mL plastic vial and the vials were then randomly allocated to the water baths. Sound and silent treatments were also randomly allocated to water baths for each experiment.

Once all megalopae were transferred to experimental treatments, the MP3 player was turned on to begin playback in a sound treatment – signifying the beginning of the experiment. Every six hours, the individual crabs were examined to determine if they had settled and metamorphosed into a first instar juvenile crab. The period from the commencement of the experiment until the appearance of the first instar juvenile in each vial was termed ‘time to metamorphosis’ (‘TTM’ [19]). When counts were made during the night, a dark red light was used to minimise disturbance of the crabs [30].

The experiment was terminated when all megalopae in all replicates for all treatments had settled and metamorphosed into first instar juvenile crabs. No mortality events occurred.

### Underwater sound recordings for playback experiments

A recording of a tidal turbine was not possible to obtain because there is only a few operational tidal turbines anywhere in the world and operators with recordings of turbines refused to supply them for this study. Thus a digital analogue, which matched the same frequency composition and peak intensities, was used for the sound treatments and was based on a published spectra of a tidal turbine operating under a maximum tidal flow of 3 ms^−1^ [11].

Underwater recordings from the Utgrunden coastal wind farm in Denmark were used during playback experiments and provided by Dr. Jakob Tougaard from the National Environmental Research Institute, Denmark.

Ambient underwater sound was recorded in February 2012 during late evening (19∶00–21∶00 hrs) chorus within a subtidal mudflat habitat in the southern arm of Kaipara Harbour where both experimental crab species are found in abundance, including large numbers of juveniles (S 36° 24′ 36.5" E 174° 22′ 40.9"). Calibrated High Tech, Inc. HTI-96 omnidirectional hydrophones (10 Hz to 60 kHz flat response) connected to a watertight temporal recording unit (20 dB gain, 16 bit, 48 kHz sampling rate) were used to record mudflat sound.

Before each experiment begun, a calibrated hydrophone (HTI-96 min, High Tech Inc. USA) was used to adjust the source level produced from the Phillips loudspeakers in each replicate sound treatment to the desired sound level (either 145 or 125 dB re 1 µPa for turbine treatments or 125 dB re 1 µPa for mudflat treatment). These levels were used because 145 dB re 1 µPa was the greatest output level achievable with the speaker and was as close as possible to the published source levels of an operating tidal (175 dB re 1 µPa [11] and wind turbine (154 dB re 1 µPa [10]). An output level of 125 dB re 1 µPa for the mudflat treatment was selected as this was the measured mean ambient sound level for that habitat during dusk in summer. An output level of 125 dB re 1 µPa for a tidal turbine sound treatment was also used in one experiment to match the intensity level of mudflat sound treatment to determine if sound level alone was responsible for influencing metamorphosis behaviour in crab megalopae (refer to table 1).

**Table 1:** Summary from seven individual experiments of comparisons of median TTM values among replicates within each treatment.

Experiment	Species	Sample size (n)	Treatment	*P*-value	H-statistic
1	*Austrohelice*	30	Tidal turbine	0.56	1.08
	*crassa*		(145 dB re 1 µPa)		
		27	Wind turbine	1.00	0.02
			(145 dB re 1 µPa)		
		30	Mudflat	0.48	1.45
			(125 dB re 1 µPa)		
		30	Silent	0.19	3.31
2	*Hemigrapsus*	21	Tidal turbine	0.43	1.67
	*crenulatus*		(145 dB re 1 µPa)		
		21	Wind turbine	1.00	0.01
			(145 dB re 1 µPa)		
		21	Mudflat	0.07	5.43
			(125 dB re 1 µPa)		
		21	Silent	0.81	0.41
3	*Austrohelice*	30	Tidal turbine	0.58	1.08
	*crassa*		(145 dB re 1 µPa)		
		30	Silent	0.33	2.44
4	*Austrohelice*	27	Wind turbine	0.10	0.02
	*crassa*		(145 dB re 1 µPa)		
		27	Silent	0.59	1.07
5	*Hemigrapsus*	30	Tidal turbine	0.43	1.67
	*crenulatus*		(145 dB re 1 µPa)		
		30	Silent	0.91	0.20
6	*Hemigrapsus*	21	Wind turbine	1.00	0.01
	*crenulatus*		(145 dB re 1 µPa)		
		21	Silent	0.81	0.41
7	*Austrohelice*	27	Tidal turbine	0.79	0.46
	*crassa*		(145 dB re 1 µPa)		
		27	Tidal turbine	0.26	2.68
			(125 dB re 1 µPa)		
		27	Silent	0.21	3.15

Kruskal-Wallis test showing no significant difference for replicates within all experimental treatments (*P*>0.05).

Unfortunately, comparisons of TTMs between experiments were not appropriate due to an inability to accurately determine the starting ages of the wild-caught megalopae. As such, a series of seven experimental combinations were necessary because of the vagaries of supply of wild megalopae. The seven experiments each tested an individual combination of experimental treatments. Comparisons among treatments were possible within individual experiments as all subject megalopae were from the same wild-caught cohort and were randomly assigned to experimental treatments and replicates.

### Data analyses

Nonparametric statistical methods were used to analyse the differences between median TTM values within and among treatments. Mann-Whitney tests or a Kruskal-Wallis one-way analysis of variance on ranks were used to test for differences in the median TTM among replicates within individual treatments (i.e., a separate analysis for each treatment). If these comparisons were not significant for each treatment, then the TTMs for all replicate tanks within each treatment were pooled and used to compare the median TTMs among the treatments (Stanley *et al*., 2010). For all statistical comparisons, a *P* value ≤0.05 was considered significant. Dunn’s pairwise multiple comparisons tests were used to determine differences in the median TTMs between individual pairs of treatments where the overall experiment had been found to contain significant differences among treatments. All statistical analyses were carried out using the statistical software Sigma Plot 11.0 and Minitab 15.0.

## Results

### Confirmation of Sound Sources

**Figure 1:**
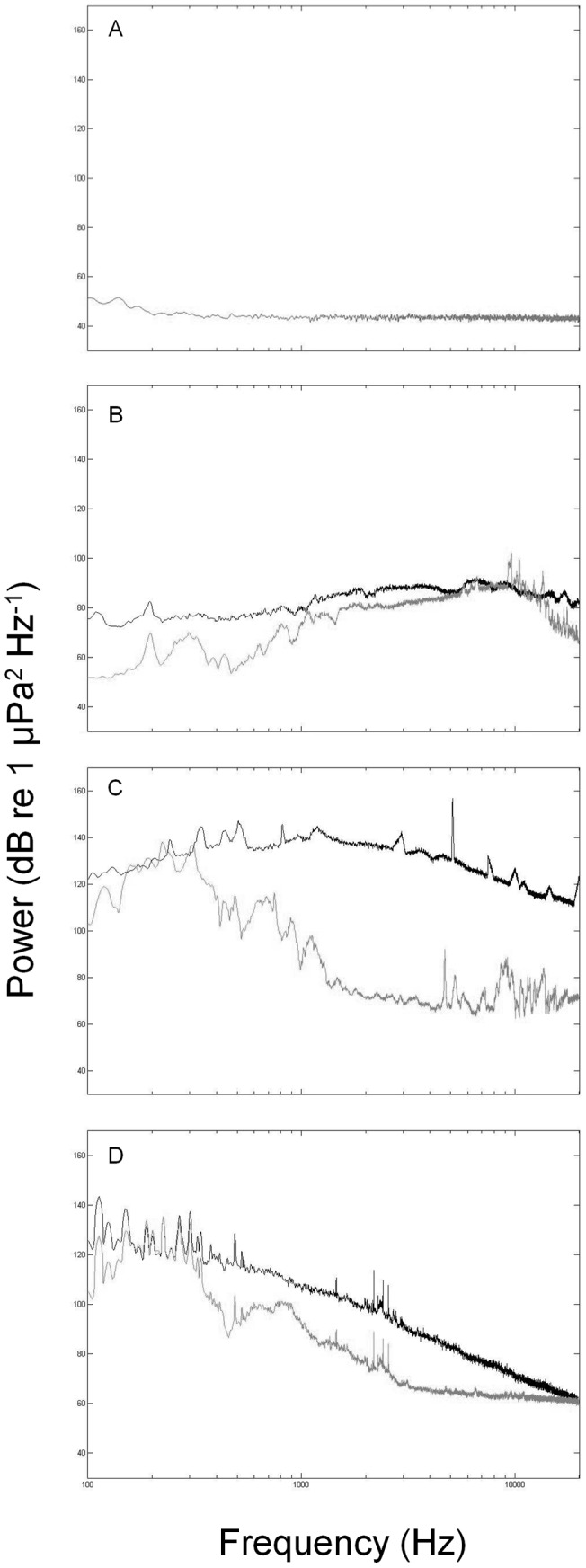
Spectral composition of experimental sound sources and when played back in experimental tanks. A) Sound recorded in silent treatment tanks (control); B) Mudflat recording from the Kaipara Harbour, New Zealand; C) Underwater tidal turbine; D) Wind turbine in coastal waters of Denmark. Black lines represent digital analogue (as in the case for tidal turbine sound) or *in situ* recordings (as in the case of wind turbine and mudflat sound).

For the wind or tidal turbine sound exposure treatments, the resulting sound in the experimental tanks was of an overall similar spectral composition to the source signals (Figure 1). Broadcasted mudflat sound replayed into replicate experimental tanks also matched the overall spectral composition and intensity of the *in situ* recording (Figure 1). Hydrophone recordings from the silent controls confirmed the absence of any sound being transmitted from sound treatments or external sources (Figure 1).

### Pooling of replicates

There was no significant difference in the median TTMs among individual replicates within both the sound and the silent treatments for all seven metamorphosis experiments (Kruskal-Wallis test) (Table 1). Therefore, in all experiments the results from individual replicates for each treatment were able to be pooled together for comparison between the pooled results from other treatments within each experiment.

### Effect of mudflat sound on the TTM

The megalopae of both crab species showed a significantly shorter median TTM when exposed to mudflat sound compared to the silent control with *A. crassa* and *H. crenulatus* showing a 31% (*H*_3_ = 29.13, *P*<0.001) (Table 2, experiment 1) and 21% (*H*_3_ = 23.23, *P*<0.001) (Table 2, experiment 2) reduction in median TTM, respectively.

**Table 2:** Comparisons among median TTM for each treatment for two estuarine crab species from seven individual sound exposure experiments.

Experiment	Species	Treatment	Median TTM	Difference from	*P*- value	*H*-statistic*
			(h)	Silent control		*U*-value**
				TTMs (h)		
1	*Austrohelice*	Tidal turbine	114	27^b^		
	*crassa*	(145 dB re 1 µPa)				
		Wind turbine	111	24^b^		
		(145 dB re 1 µPa)			<0.001	29.129*
		Mudflat habitat	60	27^c^		
		(125 dB re 1 µPa)				
		Silent control	87	0^a^		
2	*Hemigrapsus*	Tidal turbine	144	54^b^		
	*crenulatus*	(145 dB re 1 µPa)				
		Wind turbine	150	60^b^		
		(145 dB re 1 µPa)			<0.001	23.229*
		Mudflat habitat	90	24^c^		
		(125 dB re 1 µPa)				
		Silent control	114	0^a^		
3	*Austrohelice*	Tidal turbine	114	30^b^		
	*crassa*	(145 dB re 1 µPa)			0.006	234.0**
		Silent control	84	0^a^		
4	*Austrohelice*	Wind turbine	156	24^b^		
	*crassa*	(145 dB re 1 µPa)			0.04	238.5**
		Silent control	132	0^a^		
5	*Hemigrapsus*	Tidal turbine	126	24^b^		
	*crenulatus*	(145 dB re 1 µPa)			0.006	189.5**
		Silent control	102			
6	*Hemigrapsus*	Wind turbine	150	36^b^		
	*crenulatus*	(145 dB re 1 µPa)			0.04	141.0**
		Silent control	114	0^a^		
7	*Austrohelice*	Tidal turbine	132	24^b^		
	*crassa*	(145 dB re 1 µPa)				
		Tidal turbine	126	18^b^	0.025	7.348*
		(125 dB re 1 µPa)				
		Silent control	108	0^a^		

Different superscript letters indicate significant difference between median TTMs within an individual experiment (*P*<0.05).

### Effect of turbine sounds on the TTM

Both wind and tidal turbine sound at levels of 145 dB re 1 µPa caused a significantly longer median TTM in the megalopae of *A. crassa*, and *H. crenulatus*, compared to silent control treatments (Table 2, experiment 3, 4, 5, & 6).

The megalopae of *A. crassa* that were subjected to tidal turbine sound at 145 dB re 1 µPa showed an increase in TTM of approximately 26%, compared to the silent control treatment (Mann-Whitney *U* test, *P* = 0.006) (Table 2, experiment 3). The megalopae of *H. crenulatus* also showed a significant increase (19%) in TTM when subjected to tidal turbine sound compared to the silent control (Mann-Whitney *U* test, *P* = 0.042) (Table 2, experiment 4).

Compared to silent control treatments, wind turbine sound at a level of 145 dB re 1 µPa was also found to delay metamorphosis in both *A. crassa* and *H. crenulatus*, with an increase in median TTM by 15% (Mann-Whitney *U* test, *P* = 0.006) (Table 2, experiment 5) and 24% (Mann-Whitney *U* test, *P* = 0.042) (Table 2, experiment 6), respectively.

### Effect of mudflat sound versus anthropogenic sound on TTM

When *A. crassa* megalopae were exposed to mudflat sound at the same level as *in situ* mudflat sound (i.e., 125 dB re 1 µPa), the median TTM decreased by 47% when compared to the tidal turbine sound treatment, and 46% compared to wind turbine treatments (*H*_3_ = 29.13, *P*<0.001) (Table 2, experiment 1). Similarly, *H. crenulatus* megalopae showed decreases of 38% and 40% when exposed to tidal and wind turbine sound, respectively (*H*_3_ = 23.23, *P*<0.001) (Table 2, experiment 2).

### Effect of turbine sound intensity on median TTM

**Figure 2:**
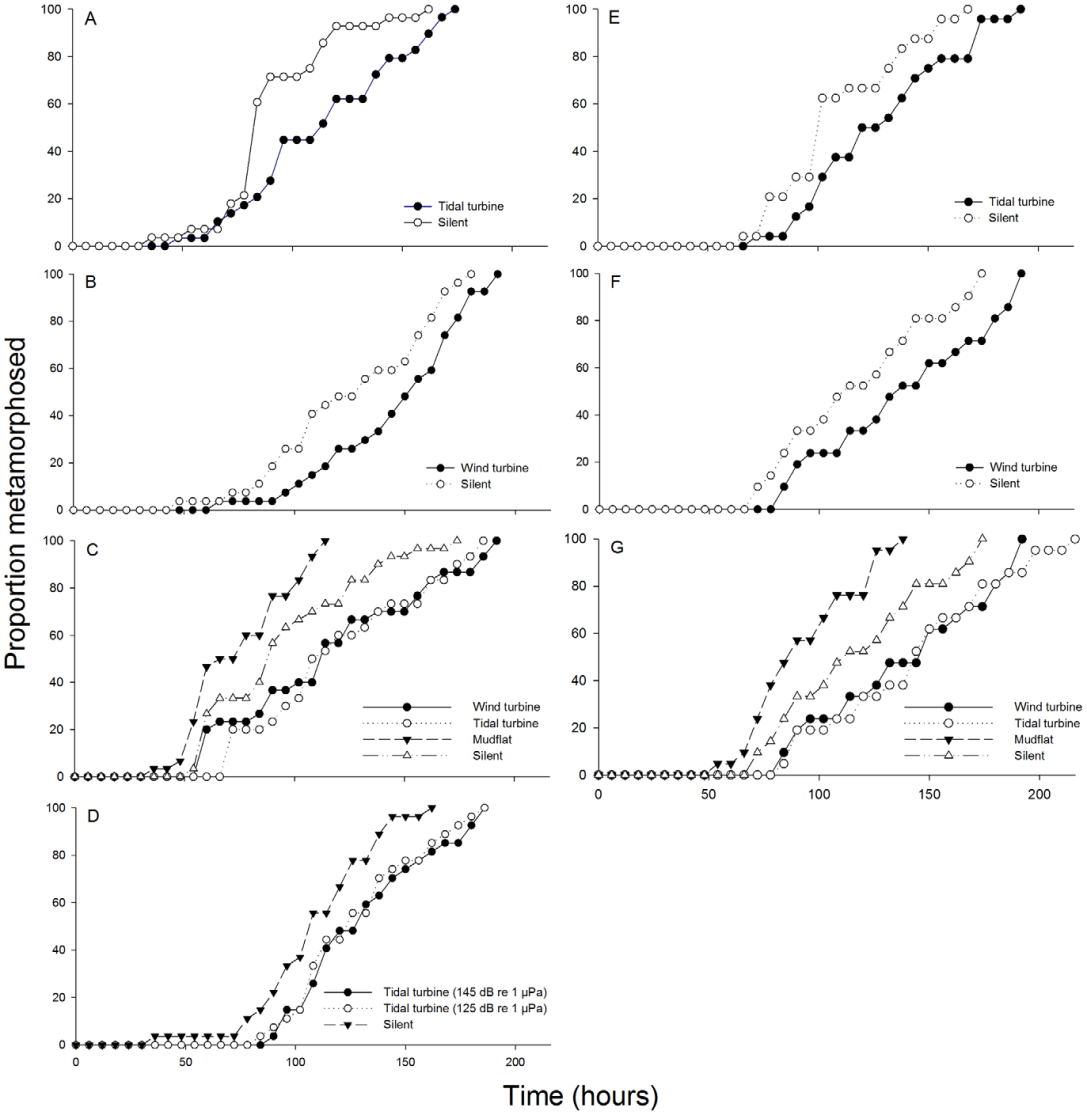
Percentage (**%**) **of total megalopae to metamorphose against time** (**hours**)**.** *Austrohelice crassa* experiments: (A) experiment 3; (B) experiment 4; (C) experiment 1; (D) experiment 7. *Hemigrapsus crenulatus* experiments: (E) experiment 5; (F) experiment 6; (G) experiment 2.

There was no significant difference in median TTM between *A. crassa* megalopae exposed to tidal turbine sound at a source level of either 145 or 125 dB re 1 µPa (Mann-Whitney *U* test, *P* = 0.69) (Table 2, experiment 7; Figure 2). However, the median TTM in *A. crassa* megalopae in both sound level treatments (i.e., 145 and 125 dB re 1 µPa) were significantly longer than the silent control by 17–22% (Mann-Whitney *U* test, *P*<0.05) (Table 2, experiment 7).

## Discussion

International interest in renewable energy production using tidal and wind turbines is growing extremely rapidly. However, there has been limited research into the environmental impact of these technologies, especially the impact of emitted underwater sound on marine life. Natural sources of underwater sound have previously been found to play an important role in influencing the settlement of many coastal organisms, including the megalopae of many coastal crab species [14], [19], as well as fish, mussel and coral larvae [17], [31], [32]. Therefore, there is the potential for the underwater sound from wind and tidal turbines installed in shallow water habitats, to interfere with these natural acoustic settlement cues. The present study found natural mudflat sound to consistently reduce the median TTM compared to silent controls by 21–31% in two crab species *A. crassa* and *H. crenulatus* which are common inhabitants of soft-shore habitats in New Zealand. In comparison, when *A. crassa* megalopae were previously experimentally exposed to underwater reef sound they showed no significant reduction in TTM compared to the silent control, which suggests that this species has habitat-specific sound cues for settlement, as have been found in other coastal brachyuran crab species [33].

Underwater sound from turbines with a source level of 145 dB re 1 µPa was found to delay metamorphosis of the megalopae of both crab species by 27–31% for tidal turbine sound and 27–32% for wind turbine sound, compared to silent control treatments. A delay in metamorphosis may prevent megalopae from settling into suitable habitats and will result in them spending more time in the plankton which is likely to increase their already high risk of predation [19], [34], [35]. This could lead to lower recruitment of crab species within estuaries and other soft-shore habitats in the vicinity of coastal turbines. Delayed metamorphosis due to underwater sound from turbines may also be an issue in any other species which have sensitivity to acoustic settlement cues, such as coral, mussels and fish [17], [31], [32], [36]. Furthermore, the interference in the metamorphosis responses in crab megalopae when subjected to varying intensity levels of tidal turbine sound may also suggest that other continuous anthropogenic underwater sound sources of similar frequency composition and intensity, such as shipping (most acoustic energy below 1 kHz [37]), may have similar effects on settlement and metamorphosis in megalopae.

While these results suggest turbine sound may mask natural acoustic settlement cues, the spatial scale over which such masking may occur is difficult to infer because little is known about acoustic detection thresholds of crustaceans. Previous research has investigated the acoustic settlement response thresholds in the megalopae of a range of brachyuran crab species and these were found to vary substantially among species [33]. For example, the megalopae of *Leptograpsus variegatus*, *Cyclograpsus lavauxi* and *Hemigrapsus sexdentatus* showed behavioural response thresholds of 90, 100 and 126 dB, respectively, to acoustic settlement cues from preferred settlement habitat [33]. Given these measured behavioural thresholds, the associated distances these crab species may be able to detect and respond to acoustic settlement cues were estimated at 199 and 39,811 m assuming spherical and cylindrical spreading of sound from the source, respectively [33]. Acoustic behavioural response thresholds for *A. crassa* or *H. crenulatus* are not known, however, if response thresholds are assumed to be similar to *L. variegatus*, *C. lavauxi* and *H. sexdentatus*, and the same cylindrical spreading and transmission loss models from past studies are applied, then the potential impact of turbine sound delaying metamorphosis could range for up to 40 km from the turbine source.

Besides acting as a settlement cue, natural sources of underwater sound from suitable settlement habitats also have a strong influence on the swimming behaviour in crab megalopae, with crabs orienting their swimming toward the sound source, presumably to assist in locating suitable settlement habitats [14]. Although not examined in this study, it seems likely that underwater turbine sound may also interfere with the orientation behaviour of swimming crab megalopae, in the same manner it has been shown to interfere with their acoustic metamorphosis response. Testing this possibility warrants further research as it has the potential to have a greater influence of the spatial distribution of settling crab larvae in relation to underwater turbines.

Poorer recruitment and subsequently smaller local populations of estuarine crabs may have ecological effects due to their extremely high abundances (i.e., over 550 m^−2^ for *A. crassa* [38], [39]), importance in bioturbation and nutrient cycling in shallow waters [40], and as a food source for many commercially important coastal fishes [41].

Metamorphosis in both *A. crassa* and *H. crenulatus* appeared to be delayed beyond the assumed temporal threshold (theoretically represented by control treatments [19] by at least 18 h when subjected to turbine sounds. This may be due to metamorphosis being delayed due to perceived unfavourable conditions, or because of an absence of appropriate habitat-specific acoustic settlement cues [42]. Since exceeding temporal thresholds are believed to be important in determining survival and subsequent juvenile development [19], investigating the juvenile growth rates, feeding behaviours and overall mortality following the metamorphosis of turbine sound treatment megalopae would also provide insight into the possible long-term ecological effects from turbine sound. Longer-term experiments would also help to establish if these crabs are capable of habituating to the anthropogenic sound.

The absence of a difference in the median TTM between tidal turbine sound intensity treatments (i.e., 125 versus145 dB re 1 µPa, expt. 7) suggests the observed delayed metamorphosis responses are more likely due to frequency composition of the anthropogenic sound rather than intensity alone, or at least a combination of both. The source levels of both turbine sounds are significantly greater than ambient sound and most of the acoustic energy resides in frequencies below 1 kHz [10] and 8 kHz [11] in wind and tidal turbine sounds, respectively. Several peak intensities are exhibited in tidal turbine sound at 0.3, 0.8, 2 and 5 kHz [11], while the wind turbine has a more even spread of intensity across frequencies. While it is tempting to speculate on differences in the spectra between the sounds of natural habitat, which induced a metamorphosis response, versus the turbine sounds which inhibited the response, the determination of these differences will be challenging.

## Conclusions

The results of the current study indicate that underwater sound produced by wind and tidal turbines have the potential to interfere with natural acoustic settlement cues in coastal crab species, most likely delaying or discouraging metamorphosis of megalopae whilst in the vicinity of the turbine. The effect of the underwater sound from turbines on crab megalopae appears to be related to the frequency composition of the turbine sound and not the intensity of the sound *per se*. Given that the underwater sound produced by turbines is of relatively high intensity compared to the ambient underwater sound typically encountered in coastal environments it is likely that the active frequencies of turbine sound has the potential to interfere with the metamorphosis of megalopae over a considerable radius around a turbine. To fully determine the impacts of turbine sound further research needs to confirm whether turbine sound will interfere with metamorphosis when combined with natural underwater sound *in situ*, and whether the orientation responses of swimming crab megalopae are also affected by underwater turbine sound. It would be useful to define the specific frequencies of underwater sound from turbines that interferes with the metamorphosis of crab megalopae, as it may provide a route to mitigate any effect of the underwater sound by adjusting the mechanics of the turbines to alter the characteristics of their sound emissions.

## References

[1] Inger R, Attrill MJ, Bearhop S, Broderick AC, Grecian W, et al (2009) Marine renewable energy: Potential benefits to biodiversity? An urgent call for research. J Appl Ecol 46: 1145–1153.

[2] King DA (2004) Climate change science: Adapt, mitigate, or ignore? Science 303: 176–177. 14715997 10.1126/science.1094329

[3] Rosenzweig C, Karoly D, Vicarelli M, Neofotis P, Wu Q, et al (2008) Attributing physical and biological impacts to anthropogenic climate change. Nature 453: 353–357. 18480817 10.1038/nature06937

[4] Herbert GM, Iniyan S, Sreevalsan E, Rajapandian S (2007) A review of wind energy technologies. Renew Sust Energ Rev 11: 1117–1145.

[5] Cada G, Ahlgrimm J, Bahleda M, Bigford T, Stavrakas SD, et al (2007) Potential impacts of hydrokinetic and wave energy conversion technologies on aquatic environments. Fisheries 32: 174–181.

[6] Ferro BD (2006) Wave and tidal energy. Its emergence and the challenges it faces. refocus 7: 46–48.

[7] Slabbekoorn H, Bouton N, van Opzeeland I, Coers A, ten Cate C, et al (2010) A noisy spring: The impact of globally rising underwater sound levels on fish. Trends Ecol Evol 25: 419–427. 20483503 10.1016/j.tree.2010.04.005

[8] Thomas G (2009) Noise profiles of other activities. In: Overview of the impacts of anthropogenic underwater sound in the marine environment.: OSPAR Commission.

[9] Lloyd TP, Turnock SR, Humphrey VF (2011) Modelling techniques for underwater noise generated by tidal turbines in shallow waters; 777–785.

[10] Wahlberg M, Westerberg H (2005) Hearing in fish and their reactions to sounds from offshore wind farms. Mar Ecol Prog Ser 288: 295–309.

[11] Parvin SJ, Workman R, Bourke P, Nedwell JR (2005) Assessment of tidal current turbine noise at the Lynmouth site and predicted impact of underwater noise at Strangford Lough. Subacoustictech Ltd. 628 R 0102 628 R 0102.

[12] Richards SD, Harland EJ, Jones SAS (2007) Underwater Noise Study Supporting Scottish Executive Strategic Environmental Assessment for Marine Renewables: QinetiQ Ltd. 06/02215/2 06/02215/2.

[13] Mann DA, Casper BM, Boyle KS, Tricas TC (2007) On the attraction of larval fishes to reef sounds. Mar Ecol Prog Ser 338: 307–310.

[14] Radford CA, Jeffs AG, Montgomery JC (2007) Directional swimming behavior by five species of crab postlarvae in response to reef sound. Bull Mar Sci 80: 369–378.

[15] Radford CA, Jeffs AG, Tindle CT, Montgomery JC (2008) Temporal patterns in ambient noise of biological origin from a shallow water temperate reef. Oecologia 156: 921–929. 18461369 10.1007/s00442-008-1041-y

[16] Radford CA, Stanley JA, Tindle CT, Montgomery JC, Jeffs AG (2010) Localised coastal habitats have distinct underwater sound signatures. Mar Ecol Prog Ser 401: 21–29.

[17] Simpson SD, Meekan M, Montgomery J, McCauley R, Jeffs A (2005) Homeward sound. Science 308: 221. 15821083 10.1126/science.1107406

[18] Simpson SD, Radford AN, Tickle EJ, Meekan MG, Jeffs AG (2011) Adaptive avoidance of reef noise. PLoS ONE 6: e16625. 21326604 10.1371/journal.pone.0016625 PMC3033890

[19] Stanley JA, Radford CA, Jeffs AG (2010) Induction of settlement in crab megalopae by ambient underwater reef sound. Behav Ecol 21: 113–120.

[20] Medina JM, Tankersley RA (2010) Orientation of larval and juvenile horseshoe crabs *Limulus polyphemus* to visual cues: Effects of chemical odors. Current Zoology 56: 618–633.

[21] Pawlik JR (1992) Chemical ecology of the settlement of benthic marine invertebrates. Oceanogr Mar Biol 30: 273–335.

[22] Steinberg MK, Krimsky LS, Epifanio CE (2008) Induction of metamorphosis in the Asian shore crab H*emigrapsus sanguineus*: Effects of biofilms and substratum texture. Estuaries Coast 31: 738–744.

[23] Forward RB, Tankersley RA, Rittschof D (2001) Cues for metamorphosis of brachyuran crabs: An Overview. Am Zool 41: 1108–1122.

[24] Gebauer P, Paschke K, Anger K (2004) Stimulation of metamorphosis in an estuarine crab, *Chasmagnathus granulata* (Dana, 1851): Temporal window of cue receptivity. J Exp Mar Biol Ecol 311: 25–36.

[25] Gebauer P, Paschke K, Anger K (1999) Costs of delayed metamorphosis: Reduced growth and survival in early juveniles of an estuarine grapsid crab, *Chasmagnathus granulata* . J Exp Mar Biol Ecol 238: 271–281.

[26] Pechenik JA (1990) Delayed metamorphosis by larvae of benthic marine-invertebrates: does it occur? Is there a price to pay? Ophelia 32: 63–94.

[27] Forward Jr RB, DeVries MC, Rittschof D, Frankel DAZ, Bischoff JP, et al (1996) Effects of environmental cues on metamorphosis of the blue crab *Callinectes sapidus* . Mar Ecol Prog Ser 131: 165–177.

[28] Weber JC, Epifanio CE (1996) Response of mud crab (*Panopeus herbstii*) megalopae to cues from adult habitat. Mar Biol 126: 655–661.

[29] Forward Jr RB, Rittschof D (2000) Alteration of photoresponses involved in diel vertical migration of a crab larva by fish mucus and degradation products of mucopolysaccharides. J Exp Mar Biol Ecol 245: 277–292. 10699215 10.1016/s0022-0981(99)00169-0

[30] Jeffs AG, Holland RC (2000) Swimming behaviour of the puerulus of the spiny lobster, *Jasus edwardsii* (Hutton, 1875) (Decapoda, Palinuridae). Crustaceana 73: 847–856.

[31] Vermeij MJ, Marhaver KL, Huijbers CM, Nagelkerken I, Simpson SD (2010) Coral larvae move toward reef sounds. PLoS ONE 5: e10660. 20498831 10.1371/journal.pone.0010660 PMC2871043

[32] Wilkens SL, Stanley JA, Jeffs AG (2012) Induction of settlement in mussel (*Perna canaliculus*) larvae by vessel noise. Biofouling 28: 65–72. 22235850 10.1080/08927014.2011.651717

[33] Stanley JA, Radford CA, Jeffs AG (2011) Behavioural response thresholds in New Zealand crab megalopae to ambient underwater sound. PLoS ONE 6: e28572. 22163314 10.1371/journal.pone.0028572 PMC3233601

[34] OConnor NJ (1991) Flexibility in timing of the metamorphic molt by fiddler crab megalopae *Uca pugilator* . Mar Ecol Prog Ser 68: 243–247.

[35] OConnor NJ, Gregg AS (1998) Influence of potential habitat cues on duration of the megalopal stage of the fiddler crab *Uca pugnax* . J Crustac Biol 18: 700–709.

[36] Montgomery JC, Jeffs A, Simpson SD, Meekan M, Tindle C (2006) Sound as an orientation cue for the pelagic larvae of reef fishes and decapod crustaceans. Adv Mar Biol 51: 143–196. 16905427 10.1016/S0065-2881(06)51003-X

[37] Wright AJ (2008). International Workshop on Shipping Noise and Marine Mammals. Hamburg, Germany.

[38] Jones MB, Simons MJ (1983) Latitudinal variation in reproductive characteristics of a mud crab, *Helice crassa* (Grapsidae) (New Zealand). Bull Mar Sci 33: 656–670.

[39] Morrisey DJ, DeWitt TH, Roper DS, Williamson RB (1999) Variation in the depth and morphology of burrows of the mud crab *Helice crassa* among different types of intertidal sediment in New Zealand. Mar Ecol Prog Ser 182: 231–242.

[40] Sivaguru K (2000) Feeding and burrowing in a North Island New Zealand population of the estuarine mud crab, *Helice crassa.* MSc Thesis, The University of Auckland.

[41] Gunson D (1993) A Guide to the New Zealand Seashore. Auckland: Viking Pacific.

[42] Stanley JA, Radford CA, Jeffs AG (2012) Location, location, location – finding a suitable home in amongst the noise. Proc R Soc B 279: 3622–3631. 10.1098/rspb.2012.0697 PMC3396902 22673354

